# The Relationship between Practitioners and Caregivers during a Treatment of Palliative Care: A Grounded Theory of a Challenging Collaborative Process

**DOI:** 10.3390/ijerph18158081

**Published:** 2021-07-30

**Authors:** Paolo Rossi, Matteo Crippa, Gianlorenzo Scaccabarozzi

**Affiliations:** 1 Department of Sociology and Social Research, Università degli Studi di Milano-Bicocca, 20126 Milano, Italy; paolo.rossi@unimib.it; 2 Fondazione Floriani, 20154 Milano, Italy; 3 Dipartimento Fragilità Rete Locale Cure Palliative, ASST di Lecco, 23900 Lecco, Italy; g.scaccabarozzi@asst-lecco.it

**Keywords:** palliative care, good death, dying process, caregiver, grounded theory

## Abstract

The possibility of coming to a “good death” is a challenging issue that crosses ethical and religious beliefs, cultural assumptions, as well as medical expertise. The provision of palliative care for relieving patients’ pain is a practice that reshapes the path to the event of death and gives form to a particular context of awareness, recalling the notion proposed by Glaser and Strauss. This decision redesigns the relationships between patients, practitioners and caregivers and introduces a new pattern of collaboration between them. Our study focuses on the implications of the collaboration between practitioners and caregivers, starting from the assumption that the latter may provide support to their loved ones and to the practitioners, but need to be supported too. We provide a qualitative analysis of this collaboration based on an empirical research that took place in four different settings of provision of palliative care, reporting the contrast between the affective engagement of caregivers and the professional approach of practitioners. We claim that this ambivalent collaboration, while embedded in contingent and incommensurable experiences, brings to the fore the broader understanding of the path to a “good death,” outlining its societal representation as a collective challenge.

## 1. Introduction

The study of the collaboration between the practitioners and the caregivers of a patient who is receiving or has received a treatment of palliative care is an emerging theme, both in the medical academic community and in the domain of social sciences [1]. The reasons that explain the growing interest in this topic can be traced back to various factors. Among these, two are particularly important for our reflection. On the one hand, the growth of a social and cultural movement, from the seventies, that claims for the acknowledgment of the “good death” as a social right [2,3]. On the other hand, the position of international medical institutions (such as the WHO) stated that the provision of palliative care should depend on need and not only on prognosis [1,4].

This means that it is eventually the patient who is entitled to accept or refuse this treatment, according to his/her desires. However, once s/he accepts this treatment, as long as s/he is aware of the incumbent process of dying, his/her will to pursue his/her desires reshapes the relations between practitioners and caregivers. The latter are particularly hit by this change: they need both to support their loved one and to be supported in this final step. Some scholars noticed that, along this process, the patient and his/her caregivers form a “unit of care” [5].

It is thus possible to argue that collaboration with practitioners is one of the most relevant stakes in the treatment of palliative care. They are both involved in the achievement of a “good death” of the patient or, as scholars pointed out, a “good enough death”, which results from an adaptation of the original concept of “good death” [6].

The concept of “good death” refers to a complex nexus of cultural, professional, and institutional issues [7,8]. The definition of this notion is a debated matter that—across a variety of questions—turns around the ambiguity and incommensurability of its adjectivation: since the patient cannot account for his/her own experience, how can we state what is a “good” death? Answering this question is a controversial challenge [8], but nowadays it has been clarified that the notion of “good death” in the palliative care framework is very different from the notion and the practices of euthanasia. In pursuing “good death”, or at least “good enough death”, caregivers and practitioners hold the responsibility for fulfilling the patient’s needs who accepted palliative care. These can refer to very different expectations. For instance, Cain and McCleskey [9] argue that the definition of the “good death” revolves around some principles that remark a set of heterogeneous social expectations: “pain relief, acceptance, mending of familial and other important relationships, and not being a burden to others” (p. 1176).

We claim that when dealing with the issue of “good death” the focus of the research on what can be considered “good” should focus on the support provided by practitioners and caregivers, rather than on the event of death. While this statement could sound tautological, we draw from this assumption the necessity of considering the collaboration between caregivers and practitioners as a challenging issue. The trajectories followed by these actors for pursuing the “good death” of the patient can be confluent and collaborative as well as misleading and contested. The aim of our study is to propose an explorative analysis of the issues that may emerge during this collaboration, focusing on the different representations that these actors can attach to their collaboration. We designed this analysis as a sort of dialogue between these actors, encountered in different settings, where the provision of palliative care follows different arrangements, enlarging the diversification of the representations attached to it.

In pursuing this goal, our article opens a new interstitial space for qualitative analysis of the event of death, bridging the broader domain of sociology of death with the more specific and scarcely explored (in the sociological literature) topic of palliative care. Moreover, this article fills a gap in the literature, proposing an analysis that crosses and combines two different viewpoints on palliative care, rather than observing the implications of this treatment standing on a binding position.

### Palliative Care and the Process of Dying: Beliefs, Conditions, Practices

A review of the literature on the sociology of death is far beyond the scope of this article [10,11,12]. We rather prospect an analysis of the pursuit of a good death through a treatment of palliative care adopting a sociological approach, with the purpose of emphasizing its construction as a collaborative endeavor promoted by practitioners and caregivers. Our analytical framework intersects two viewpoints: firstly, as the background of our analysis, we reflect on the institutional status of palliative care; secondly, standing at a micro-level, we focus on the dynamics of collaboration between caregivers and practitioners.

The provision of palliative care has taken the status of an institutional matter, since it has been claimed as a social right by some movements, as the promoters of the diffusion of hospices [2,3]. This poses a twofold question: on the one hand, as long as palliative is expected to “lead” to a good death, it is important to reflect on the overall social and cultural acceptance of this claim [10,13]. On the other hand, whether the provision of palliative care is considered as a social right, it is necessary to identify the institutional conditions that ensure equal opportunities of access to this treatment. These questions are the two sides of the same coin since the definition of the institutional conditions for the provision of palliative care outlines the ethical and cultural acceptance and legitimacy of palliative care. Wegleitner, Schuchter and Prieth [14] state that the above mentioned foundational principles of palliative care express the current prevalent ethical concerns in coping with the event of a death in the western world, while they may be questioned in other cultures [15,16,17,18,19]. Timmermanns proposes the notion of “death brokering” [2] (p. 993) to illustrate the “negotiation of culturally appropriate deaths” [ibidem]. The institutional status of palliative care affects the organization of healthcare systems, whereby palliative care find different degrees of legitimacy and forms of provision [20].

The inequality of access to this treatment is one of the most evident indicators of the different values attached to palliative care. Several scholars [4,21] have pointed out the factors that hinder its provision. In spite of a growing acknowledgement of palliative care at a macro and global level [22], prompted by international organizations, it is important to report the persistence of some biases against this treatment at a professional level. One of these biases deals with the belief that a treatment of palliative care represents a failure of “traditional” medical care [23,24]. However, the persistence of this bias has been contested [25,26]. Scholars have noticed that, rather than maintaining skeptical positions from a professional point of view, physicians and clinicians are increasingly aware of having to deal with the different beliefs of patients and caregivers about the provision of palliative care [18].

The decision of providing treatment of palliative care enacts a process that calls in action several actors: practitioners (physicians, clinicians, nurses, psychologists, as well as social workers) and caregivers, who are generally informal caregivers [27] (p. 154). However, their role within the treatment is ambivalent. On the one hand, they can help to provide support both to the patient and to the practitioners. From this point of view, the interplay between practitioners, caregivers and patients can be understood through a holistic approach, emphasizing the dynamic forms of mutual support they can enact [ibidem]. On the other hand, caregivers need a peculiar support, since they have to cope with a situation they know to be irreversible. From this point of view, as argued by Fleming and colleagues, patient and caregivers constitute a single “unit of care” in a path of palliative care [5]. As stressed by the same authors, during the treatment, “the caregiver becomes at once an autonomous extension of the patient, an integral partner in the patient-physician relationship, and consequently an important member of the medical team involved in treating that patient” [5] (p. 409).

On the one hand, in his/her interactions with the practitioners, the caregiver is somehow expected to “help” the professional staff. The help provided by caregivers is manifold. Docherty and colleagues noticed that caregivers can “be more accurate in estimating patient experience when encouraged to imagine the patient’s feelings” [27] (p. 166). Caregivers can also help practitioners (and themselves) to activate a helping network [28] (p. 177). Guberman and colleagues point out that caregivers can help practitioners in dispensing complex practical tasks, such as IV treatments, pain control, feeding by tubes, wound treatment, catheters [29] (p. 249). They stress, however, that this help can be considered as a “transfer of care to families” [ibidem], as also argued by Levine and colleagues [30]. In some cases, this shift may occur taking for granted that caregivers are ready to help both practitioners and patients [31]. Still, this collaboration enacts and implies a learning path, whereby caregivers may improve their skills in performing technical activities but, alongside, are increasingly burdened by feelings of anxiety, fears of being inadequate, stress and sadness [32,33,34].

On the other hand, it is thus clear that caregivers need a peculiar support. Several studies have discussed the stress and the deterioration of the psychological condition of the caregiver [32,35,36,37]. Many studies focused on the practices that can alleviate caregiver’s burden [38,39,40,41]. The notion of burden is here intended as a multidimensional construct that includes the psychological condition of the caregiver, the difficulties that may arise at the workplace and in work-life balance, the risks of social isolation, as well as the economic costs of caregiving [42] (p. 803).

The development of a collaboration between caregivers and practitioners remains a very complex and controversial issue. One of the most challenging aspects of this relation deals with the construction of a path for leading the patient to a good death. From the viewpoint of practitioners, during this process caregivers can be a source of help and likewise a matter of concern. For the above-mentioned points, caregivers can provide a relevant support to practitioners. However, van Staa and colleagues notice that “death and dying do not emerge as a major source of stress in palliative care” [43] (p. 94). One of the elements that can stress care relationships and are often underestimated is the incomplete alignment between the needs and desires of the patient and the family [44]. Since palliative care is aimed at both the patient and his family, the ability to operate within these tensions is one of the skills needed in palliative care. Mota Vargas and colleagues emphasize that this struggle can have different implications: on the one hand, practitioners can develop a greater empathy but, on the other hand, this can lead practitioners to work overload and expose them to the risk of burnout [45].

## 2. Materials and Methods

This research is part of a broader study, Demetra 1, focused on the analysis of the implications of treatments of palliative care for the familiars and the caregivers of the patients [46]. The research has been promoted by Fondazione G. Berlucchi onlus and Fondazione Floriani, two of the leading actors of the movement for the diffusion of palliative care in Italy. Since the collaboration between caregivers and professionals in palliative care is an essential element of the quality of care processes, but at the same time it is a delicate and rarely explored issue in Italy, we adopted an inductive approach [47], developing a qualitative analysis. Thus, rather than having pre-established hypotheses to be tested, we chose to provide an explorative view on this topic, zooming between local experiences and the wider institutional and professional settings of palliative care provision in our country.

In accordance with the broader project Demetra 1, the research was conducted on the four different research units across Italy. Each context marks a territorial network of provision of palliative care. It is important to remark that the configuration of these networks is different. The main differences refer to:

(a) the central unit of the network: in three networks it was a public hospital, while in the remaining it was a different healthcare provider (a not-for-profit organization coordinating the provision of palliative care in the territory)
(b) the presence of professionals: all networks included medical practitioners (clinicians, physicians and nurses), while the presence of other practitioners (psychologists, social workers and so on) within the network was different
(c) the settings: the networks worked in and with different modalities and settings (hospitals, hospices, home)
(d) the institutional composition: each network includes different types of organization (public, private, third-sector organization), combined in different ways

Since one of the major issues in the study of palliative care deals with the difficulty of recruiting caregivers, we adopted an inclusive approach, welcoming all the caregivers who replied to and accepted our call.

Following a convenience sampling design, each of the four networks contacted, face-to-face or by telephone were the caregivers of the patients they assisted in the last 12 months, asking them to participate to focus groups. All the caregivers were relatives of the patient they assisted. The number of caregivers who participated in the focus groups is reported in Table 1. The focus group with caregivers followed a structured scheme. The moderators, after explicated the aim of the Demetra 1 project and the specific goals of the focus group, introduced four main topics, through open-ended and concise questions:

− Who suggested the treatment of palliative care?
− What did you expect from this treatment?
− How was the relation with practitioners? Did you encounter some troubles in collaborating with them?
− Could you identify the benefits you, as caregivers, enjoyed by this treatment?

In the meanwhile, each of the four networks recruited the professionals who were closer to the patient and his/her caregivers during the treatment, asking them to participate in a dedicated focus group. The criteria adopted in recruiting was to create a group of professionals involved in care pathways, with at least representations of physician and nurses. Where possible, psychologists and social workers were included in the sample. The number of participants in the focus groups of professionals is reported in Table 2.

Within each research unit, the focus group with the professionals was held after the focus group with caregivers.

The focus groups with practitioners maintained an open structure. The researchers reported the reactions of caregivers to our questions and the encounters were mainly focused on the discussion of the insights drawn from the caregivers.

P. R. conducted and M.C. acted as a facilitator in the eight focus groups carried out. Both researchers are experienced in qualitative techniques, graduated (MD) and obtained a PhD in Sociology and Methodology of social research.

Researchers did not have any previous relationship with any of the caregivers interviewed. M.C., as a part of Demetra 1 research team, had the opportunity to previously meet some of the professionals involved in the focus groups in the context of the other research activities included in the Demetra 1 protocol.

Focus groups, both with caregivers and professionals, have been held in the facility of the palliative care services, they have been audio-recorded and M.C. made field note. The study has taken place in the period between April 2018 and January 2019.

As stated earlier, the data collected from the focus groups have been analyzed following an inductive approach, adopting a grounded-theory (GT) methodology. This methodology drives the researchers to analyze data while collecting them [48,49,50,51]. In a nutshell, the basic operation of this technique is the identification of “portions” of a document (the transcription of an interview, a verbatim, field notes and so on) with the purpose to cluster them into synthetic categories.

Within the plethora of approaches GT [52], we followed the methodology proposed by Thornberg and Charmaz [48], that emphasizes the technique of focused coding. The authors suggest making constant comparisons between the different incidents, contexts and people who are involved in an instance of a broader social phenomenon. This fits with the peculiar design of our study: on the one hand, we had a fixed pattern of data collection (the questions proposed to the caregivers), while on the other hand, the heterogeneity of the territorial networks allowed us to observe and compare very different patterns of collaboration between caregivers and practitioners. However, the fixed structure of the empirical research limited the process of theoretical sampling: since we were expected to ask the same questions to the caregivers, we could not come back to these actors for deepening our understanding of specific themes. Likewise, we had just one opportunity to discuss with the practitioners of each network. Nevertheless, each discussion with the practitioners (since it followed an open track) could benefit from the findings and analyses conducted after the previous focus groups. Thus, when we arrived at the fourth visit, our knowledge of the collaboration between practitioners and caregivers had significantly raised and we could propose to formers richer insights and develop a much more informed discussion. This path allowed us to elaborate on the constructs and interpretive concepts with a growing level of abstraction.

P.R. analyzed the transcripts of the focus groups with NVivo 12. Firstly, we examined separately the data collected from the focus groups with caregivers and the data collected from the focus groups with practitioners. We later combined the analysis of these data moving to a more focused coding. In this way, we produced a sort of “dialogue” between the voices of these actors, observing the dynamic of collaboration from both sides. The quotations presented in the Results are identified by participant number according to the codes reported in Appendix A.

## 3. Results

It is possible to argue that the interpretation of the collaboration between the caregivers and the practitioners follows two partly convergent paths. On the one hand, most caregivers consider the collaboration with practitioners as an effective and collective engagement, rather than the mere provision of medical treatment. On the other hand, practitioners reply that the collaboration with caregivers is a part of their job and needs to be interpreted as a professional activity, rather than a mission (they refuse to be considered “angels”). Although they cannot completely deny the possibility of an effective involvement in the relations with the caregivers, they claim that this has to be considered as a professional commitment, rather than the expression of personal traits that may prompt empathy and closeness. Moreover, they emphasize their belonging to an emergent community that crosses the boundaries of professional sectors and is acquiring growing institutional legitimation.

**Figure 1:**
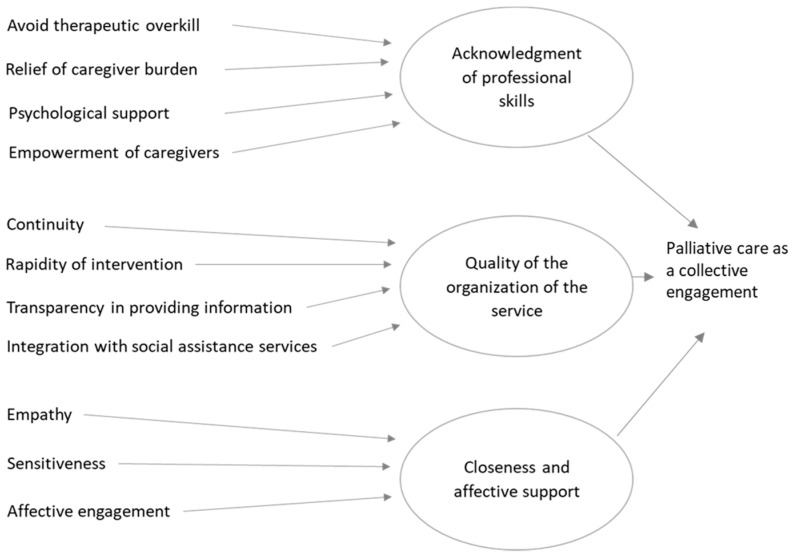
The structure of data collected from the focus groups with caregivers as emerged in our analysis.

**Figure 2:**
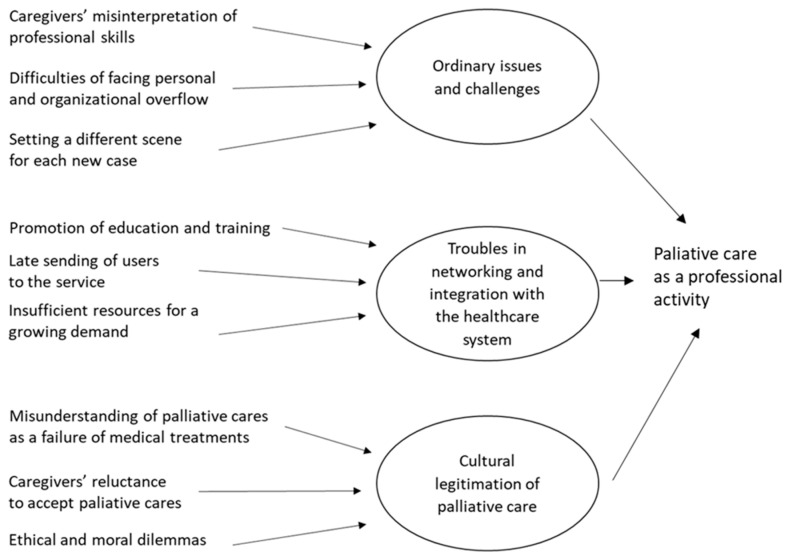
The structure of data collected from the focus groups with professionals as emerged in our analysis.

These themes represent the core categories of the two streams of analysis. They reproduce the intrinsic tension of an entangled collaboration that is intense and incommensurable, as well as challenging and controversial. Figure 1 pictures the path and the structure of the analytical process of the data collected from the focus groups with caregivers, while Figure 2 reports the path and the structure of the analytical process of the data collected from the focus groups with practitioners.

### 3.1. In the Eyes of the Caregivers: Palliative Care as an Effective and Collective Engagement

Most of the caregivers who participated in the focus groups described the collaboration with the practitioners as a collective and effective engagement. From their viewpoint, this collaboration took the form of a collective endeavor since it implied an active collaboration with the practitioners, through which they stepped to a new form of caregiving, learning how to assist their loved one in the final stage of farewell. Most of them pointed out that before the treatment of palliative care they felt to be supporting the patient nearly solitarily, while after that they felt to be engaged in a collective effort. This point emerged several times during the focus groups, as reported in the following excerpts.

*‘Yes, they all (the social worker, the psychologist, the oncologist, the nurses) were all truly impeccable, appropriate to the circumstance. I found great help, we found support at an emotional and organizational level. We also learned the type of disease my father had, how to interact, how to deal with it’.*

(P2f)

*‘I have to say they were very close to me, even when my sister was sedated. They explained to me and my nephew the whole procedure, they were very, very careful also psychologically, they helped us a lot’.*

(Fo4)

Another standing point of this collaboration was the perception of an effective involvement of practitioners. Caregivers were helped for coping with the progressive worsening of the conditions of their loved ones. Such support was not expected, especially since in the earlier stages of care the relationships with professionals were much colder and detached. The next excerpts clarify this point:

*‘Beyond the very high professionalism, which I expected, I found a great level of humanity and psychological support not only for the patient, because obviously that is fundamental, but also for us, for the family. When the situation worsened, they came to explain [to] us what were the next stages of that dramatic situation’.*

(Fo3)

*‘I think there is a big difference with what we experienced before, because before we were accustomed to interact with doctors highly trained but very cold, who treat you at times like a number. Instead, we were supported (in the palliative care unit, we were assisted psychologically. You notice that behind this there is a preparation for the psychological support, which is priceless’.*

(Fo5)

*‘I thought they were just coming to see, something like that, to provide drugs. No … they supported us at a psychological level, and they were always available: they did much, much more than I expected. Staying at home for two and a half months alone with a terminal patient, with a doctor who comes and goes, is not being supported, while they supported us’.*

(Fo4)

These notes reinforce the assumption that patients and caregivers form a “unit of care” and that the practitioners are called to support them simultaneously. During and across the focus groups, caregivers often argued that, based on their experiences, palliative care is something more than medical treatment. This was remarked when discussing the organizational aspects of the support provided by the practitioners. As caregivers realized that the time was going over, the reactivity of the service, together with its continuity and flexibility, helped them to assist the loved one without missing time. Likewise, the caregivers highlighted the ability of practitioners to reconfigure the setting of care. Most practitioners, according to caregivers, were able to set up a new “scene” for supporting the patients. This happened when a patient was moved to a hospice, as well as when the care was provided at home.

*‘The activation of the care was very fast, it lasted around ten days. They made an interview, they provided us a report, and after a week they started’.*

(L6)

*‘I am truly grateful to the doctors, the nurses, all of them. Whatever problem I had I phoned them, they answered me at any time and intervened in a very short time’.*

(Fi3)

*‘When the oncologist told me: “Look, he has 6 months left, with palliative care he won’t suffer particularly.” I feared the opposite. The Palliative Care doctor told me that he wasn’t going to discuss the diagnosis but he reassured me. He told me “I’m not going to investigate, of course, but if something is going wrong call me if it is the case we intervene”. Before we had to go through every week the same sequence: going to the laboratory, make an exam, waiting for its result… I felt growing anxiety, day by day, because we didn’t know what would happen the days after, whom we would have to speak to, and so on’.*

(Fi3)

When analyzing these data, we always reminded us that those caregivers shared a positive impression of the service of palliative care. It is also necessary to consider that the high level of quality of the service, as reported by the caregivers, can maybe be found only in some selected units. However, it is possible to argue that entanglement of professional skills, organizational flexibility, and personal commitment represents for the caregivers the peculiar added value of palliative care. These factors can enact a collaborative path, along which caregivers and practitioners are engaged in a collective endeavor.

### 3.2. A Professional Activity, Rather Than a Mission

Each focus group with the practitioners took place after the focus group with caregivers. Practitioners could thus react to the opinions expressed by the caregivers and propose different interpretations of the issues raised by them. This offered the practitioners a double opportunity. On the one hand, they could get an account of their work coming from the caregivers they “worked” with. On the other hand, they had the opportunity to discuss reflexively and collectively the sense of their work and the perception of palliative care.

Practitioners complained that caregivers, while truly appreciating their efforts, had a distorted perception of their work. One of the most common marks of this misinterpretation was the description of practitioners as “angels” who helped the patients and the caregivers to cope with a dramatic event of life.

*‘The thing that needs to be said is that the thing that bothers us the most, in the end, is being recognized as angels. That’s something that bothers the whole team a lot. (…). It bothers us a lot, because the important thing for us is that we want to be recognized as professionals. So, when so many times, caregivers ask us: “but, how do you do it?” I think this is my work’.*

(MDF1)

*‘This is a very important thing, on which there is still a lot of work to be done: the palliative care team is not made by angels, as often it looks like, but is made by professionals. In my opinion, this would be the most important and greater recognition for all of us’.*

(MDF1)

This misinterpretation may be motivated and understood considering the contrast between palliative care and other therapies provided to terminal patients. This statement is shared by caregivers and practitioners since a palliative care unit is expected to provide a closer and “tailored” support to the patient and the caregivers, as stressed by many practitioners.

*‘Why do they call us angels? Angels with respect to whom? I think, in their views, we are perceived as angels when compared to what they experienced before’.*

(INFP1)

*‘Caregivers are happy to be listened [to] … we talk, we listen, we answer them and this seems an extraordinary thing… Angels? No, it’s ordinary stuff, daily work: listening to the needs, and trying, where you can, to buffer, to help them. This is what we do’.*

(INFP4)

Likewise, practitioners fear that the caregivers might perceive their work as a mission or a vocation, rather than the “mis-en-scene” of a professional expertise. Because of this belief, the complexity of their job appears to be hidden. This may lead to forgetting their long and intensive training, both as students and as professionals in the field. Such a misunderstanding emerges in different situations. One of the most evident is the arrangement of the access of patients that is often described as a process of “check-in” by some caregivers.

*‘When patients enter the hospice, in the first interview with family members we ask them what they think about it, because there are some caregivers who say: absolutely not. After that, we try to illustrate the path, before moving to the analysis of the specific situation of the patient. Their understandings often depend on the conditions of the patient, because the evolution sometimes is so rapid that this communication needs to be very fast: so you explain in detail what it involves, what are the implications (…) because not all the families always accept the fact of not being able to have direct communication with the patient. So they struggle: “Could s/he does not wake up after? », and you have to tell «No, s/he may not wake up”’.*

(MDFo2)

In such a situation, practitioners set the scene for their activities [53], informing and involving family members and caregivers. Thanks to their expertise and experience, practitioners have been able to convert this critical process into a professional routine. This helps them to deal with the emotive implications of their daily work. Vice-versa, caregivers who cope with this experience once in their life (with the exceptions of those who already cared for a loved one) observe just the surface of this path, ignoring its professional and organizational background.

Beyond the controversial relationship with caregivers, some practitioners who participated in the focus groups argued that the value of palliative care is still under-evaluated within the medical community. Several factors can bring about this problem. Firstly, palliative care has only recently spread and is still partially accepted (if not refused) by some physicians. Older physicians are overtly accused of ignoring palliative care:

*‘A lot of elderly doctors have no idea of palliative care, whereas now, the training of General Practitioners generally provides for at least two months of study or internship. There is an inverse relation between the proximity to retirement and the propensity to palliative care’.*

(MDF2)

This statement sheds light on the larger problem of the legitimation and accreditation of palliative care. From their point of view, practitioners argue that palliative care represents a challenge also for the healthcare system as a whole.

*‘It is possible to say that there are two worlds. The smaller community of experts of palliative care and the broader medical community. Within the latter, it is possible to find practitioners who question the value of palliative care, institutions who do not listen to us, and hospitals who does not talk our language… But the world of palliative care is growing and enlarging. This is a good thing, because we learnt to talk to each other, to speak the same language. Unfortunately, there are still some problems with the rest of the world. We all witness this issue: those who have greater experience in this field, those who come from the world of reanimation wards or intensive care units… Everyone who joins this world realizes that this is a different world. We have a very strong need, a cultural need in the broadest sense: presenting an acceptable way of good dying, at all levels: social, institutional, in all sectors. And, of course, we cannot limit ourselves to doing what we have done so far: when a person or a family accesses our service, that is the moment when we do not only provide care, but we convey the ‘culture of good dying’.*

(MDP7)

As reported in the last excerpt, practitioners face different difficulties at different levels: they have to tackle institutional and cultural resistance, as well as organizational problems. One of the most relevant is the relationship with other local providers of healthcare services. When some actors (GPs, hospital doctors, managers of local healthcare services) do not cooperate with them, patients frequently access the palliative care services too late or without proper information about how they will be assisted.

*‘Many times the patient already arrives almost in a coma, I do not say dying, but unfortunately, the clinical conditions are such that at home s/he can no longer be assisted (…). In these cases, the operative center of palliative care calls us and moves the patient to our facilities (…). According to me, we should succeed to create a network of home palliative care between home services, hospices and hopefully also hospital, in order to create a unit for avoiding delays’.*

(MDP2)

## 4. Discussion

With respect to the findings of our analysis, we pointed out two partly diverging positions about the collaboration between the caregivers and the practitioners who provide a treatment of palliative care. Practitioners claim palliative care has to be considered as a professional activity, rather than a mission. They also argue to be part of a growing community that is emerging at a professional and at an institutional level, challenging old biases and obstacles. Vice-versa, the caregivers emphasize the affective commitment of practitioners, somehow considered solely as the expression of individual personal traits instead of being an essential part of the professional skills. However, this might be considered a critical issue, it could provide some insights for the education of practitioners: distinguishing the borders between professional expertise and personal engagement is a topic that should be addressed and discussed within the educative programs.

Caregivers also remark the differences between the local palliative care units and the arrangement of other types of care earlier received by their loved one, stressing the more empathic and participative approach of the service of palliative care. This fosters their perception of being involved in a collective endeavor, as a part of a care pathway that enriches the traditional doctor-patient relationship [54].

Regardless of these diverging and misleading positions, it is important to point out that the collaboration between caregivers and practitioners “brings to the fore the wider relational scene of which the phenomenon is part” [55] (p. 604). The development of this collaboration brings out the meaning attached to the notion of “good death” by the actors. However, our study suggests that this meaning is not immutable and dogmatic. All along the treatment of palliative care, this collaboration enacts a peculiar context of awareness [51] and this shapes the path towards the pursuit of a sufficiently appropriate process of dying. This collaboration is hence a challenge for both the caregivers and the practitioners. Starting from different positions, they are called to converge to a final destination, supporting each other in fulfilling the needs of the patients, hopefully sharing his/her actual wills.

Late activations, insufficient integration between the different services, lack of support for caregivers and poor information on palliative care represent obstacles to the potentiality of the treatments themselves that need to be addressed. Clinical models of palliative care seem inadequate to addressing alone the multiple co-morbidities and access issues characteristic of palliative care [56]. Advance care planning (ACP) has been described to have an important role in arouse care preferences, facilitate family communication and enable a shift of care focus towards palliative care [57,58,59]. Furthermore, a public health approach in palliative care through enhancing compassionate communities may improve legitimation of palliative care services, spread the culture of caring for people in need of palliative care and increase connections between multiple sources of help (health, social, psychological, financial) widespread on the territory, but not very connected to each other [56,60,61].

On the basis of this study, we claim that the collaboration between caregivers and practitioners is an issue that needs to be understood and developed adopting a multi-disciplinary framework. Together with medical and clinical understandings, it is important to consider the broader institutional arrangement of the path to a good death, as well as its contingent and peculiar setting in specific environments.

## 5. Strengths and Limitations

As for most qualitative research, findings cannot be generalized and we do not obviously aim at achieving this goal. Aside from this, a major issue limits our study. The caregivers who participated in the research appreciated the treatment of palliative care provided to their loved one. We did not exclude any caregivers (between those who had been supported twelve or fewer months before the focus groups) in the recruitment, but we were aware that a high number of them would have refused to participate. Thus, it is important to remark that this study is affected by a positive bias towards palliative care. According to Hudson [62], recruitment in palliative care to carry out focus groups can be problematic. Adopting a mixed method could provide different insights involving those caregivers reluctant to take part in focus groups. An anonymous questionnaire or phone interviews performed by independent entities, for instance academic institutions, could overcome selection bias.

## 6. Conclusions

In our study, we detected a tension between representations provided by the caregivers, which tend to describe the skills of the palliative care specialists in terms of personal virtues or traits, and the self-representation of the members of the palliative care teams, which emphasize the professional contents of their activities. Palliative care, especially home care services, is a matter of cooperation between patient, palliative care team and caregivers. Collaboration between practitioners and caregivers is essential to the quality of the care and to meet the needs of the patient. Caregivers may provide support to their loved ones and to the practitioners but need to be supported too. Palliative care professionals demonstrate to have a deep understanding of the burden of caregivers. In fact, one of the aspects emerged from the focus groups with practitioners is the multidimensionality of this burden. Alongside issues of a purely health nature, problems related to health care support (hygiene, preparation of meals, etc.), financial and emotional, are represented as in strong growth.

Among the different solutions that can be implemented to sustain caregivers and to encourage collaboration between the latter and the palliative care teams, adopting best practices in ACP and fostering training activities may ameliorate the already good quality of care.

Nevertheless, the ambivalent collaboration between caregivers and practitioners, while embedded in contingent and incommensurable experiences, brings to the fore the broader understanding of the path to a “good death”, outlining its societal representation as a collective challenge that needs a public health approach.

## Figures and Tables

**Table 1:** Number of caregivers who participated to each focus groups.

Location of Each Focus Group	Number of Participants	Duration	Collection Period
Lecco	6	110 min	April 2018
Firenze	7	80 min	June 2018
Forlì	7	87 min	September 2018
Palermo	11	85 min	January 2019

**Table 2:** Number and professions of practitioners who participated to the focus groups.

Location of Each Focus Group	Number of Participants	Practitioners’ Profession	Duration	Collection Period
Lecco	6	4 physicians, 2 nurses	81 min	March 2019
Firenze	8	6 physicians, 1 nurse, 1 psychologist	106 min	June 2018
Forlì	4	2 physicians, 2 nurses	57 min	September 2018
Palermo	13	7 physicians, 4 nurses, 2 social workers	105 min	January 2019

Note: See Appendix A for more detailed information about the coding of the actors.

## Data Availability

The data presented in this study are available on request from the corresponding author. The data are not publicly available due to privacy policy.

## Appendix A

We developed a coding pattern to organize the excerpts, for tracking the type of actor and the location of the focus group. The first 2 or 3 letters indicate the role of the actor (CG = Care giver; MD = Physician; N = Nurse; SW = Social Worker; PSY = Psychologist), the next letter the location of the focus group (P = Palermo; L = Lecco; F = Firenze; FO = Forlì) and the last char the number of the participant who is being quoted. For instance, “CGP2” stands for the interview to the second (2) caregiver (CG) held in the focus group in Palermo (P). The identity of each participant is hidden and cannot be recognized.
